# Lichen Depsides and Tridepsides: Progress in Pharmacological Approaches

**DOI:** 10.3390/jof9010116

**Published:** 2023-01-14

**Authors:** Isabel Ureña-Vacas, Elena González-Burgos, Pradeep Kumar Divakar, María Pilar Gómez-Serranillos

**Affiliations:** Department of Pharmacology, Pharmacognosy and Botany, Faculty of Pharmacy, Complutense University of Madrid, Plaza Ramón y Cajal s/n, 28040 Madrid, Spain

**Keywords:** depsides, tridepsides, pharmacological activities, lichens

## Abstract

Depsides and tridepsides are secondary metabolites found in lichens. In the last 10 years, there has been a growing interest in the pharmacological activity of these compounds. This review aims to discuss the research findings related to the biological effects and mechanisms of action of lichen depsides and tridepsides. The most studied compound is atranorin, followed by gyrophoric acid, diffractaic acid, and lecanoric acid. Antioxidant, cytotoxic, and antimicrobial activities are among the most investigated activities, mainly in in vitro studies, with occasional in silico and in vivo studies. Clinical trials have not been conducted using depsides and tridepsides. Therefore, future research should focus on conducting more in vivo work and clinical trials, as well as on evaluating the other activities. Moreover, despite the significant increase in research work on the pharmacology of depsides and tridepsides, there are many of these compounds which have yet to be investigated (e.g., hiascic acid, lassalic acid, ovoic acid, crustinic acid, and hypothamnolic acid).

## 1. Introduction

Using the traditional definition of lichens, these organisms are a symbiotic association consisting of a mycobiont (Ascomycota and Basidiomycota phylum) and a photosynthetic partner (which is an algae or a cyanobacterium). The rise of “Omics” technologies such as genomics, transcriptomics, proteomics, and metabolomics allows us to deeply study the symbiotic partnership in lichens. Photobiont and mycobiont are not the only members of this symbiosis. Specific bacterial microbiomes, such as Alphaproteobacteria communities and lichenicolous fungi, have also been identified and characterized on lichens [1,2,3]. Between 17,000 and 20,000 lichen species that inhabit diverse ecosystems have been identified [4]. The nutritional uses as food or flavoring agents, the spiritual uses in religious ceremonies, the industrial uses as natural dyes, and the environmental ones as biomonitors of pollution have made lichens a significant resource for different economic activities [5]. Like higher plants, lichens have been used for their therapeutic properties in many traditional medicine systems, such as those of Ayurvedic and Unani medicine, for, e.g., bronchitis, asthma, amenorrhea, stomach disorder, and vomiting [6]. For example, the *Usnea* species has been used in many cultures around the world for its antiseptic, wound healing, antibacterial, and anti-inflammatory properties. Traditional knowledge and confirmed activity studies led to the improvement of its pharmacological potential by new pharmaceutical formulations. Popovici et al. have developed bioadhesive oral films with *Usnea barbata* extract in Canola oil as an effective oral formulation [7]. However, the usefulness of lichens in the pharmaceutical industry goes far beyond this. The biological synthesis of nanoparticles (NPs) has become an active line of research. The reducing or stabilizing capacity of different natural sources, including lichens, is used in a simple, non-toxic, eco-friendly process known as green synthesis [8]. Lichenan from *Usnea longissima* was used to decorate selenium nanoparticle surfaces, showing great stability and strong free radical scavenging [9]. *Protoparmeliopsis muralis* lichen aqueous extract was used in the green synthesis of Ag, Cu, TiO_2_, ZnO, and Fe_3_O_4_ nanoparticles with antibacterial, antibiofilm, antiquorum sensing, antimotility, and antioxidant activities [10].

Furthermore, to overcome the disadvantages of this process, new biomechanochemical methods are being studied. The powdered lichens *Xanthoria elegans*, *Cetraria islandica*, *Usnea antarctica*, and *Leptogium puberulum* optimize the synthesis process of silver nanoparticles in a solid state synthesis [11].

All of these bioactive properties are attributed to their secondary metabolites. There have been around 1000 different secondary metabolites identified; these are mainly unique to these organisms and include, most notably, polyketides [12]. Three biosynthetic pathways are involved in the formation of these metabolites, such as the shikimic acid pathway for pulvinic acid derivatives; the mevalonic acid pathways for terpenes and steroids; and the polymalonate pathway, from which the main interesting groups of secondary metabolites, depsidones, depsides, and dibenzofurans are synthesized [13]. These compounds comprise from 5% to 20% of the dry weight of the thallus [6].

The present review focuses only on depsides and tridepsides and outlines the research on their bioactivities. These compounds have been identified in different lichens, such as *Pseudevernia furfuracea* (L.) Zopf (e.g., olivetoric acid); *Umbilicaria hirsuta* (Sw. Ex Westr.) Hoffm. (e.g., gyrophoric acid); *Thamnolia subuliformis* (Ehrenb.) W.L. Culb. (e.g., baeomycesic acid); *Usnea diffracta* Vain (e.g., diffractaic acid); and *Cladia aggregata* (Sw.) Nyl. (e.g., barbatic acid) [14,15,16,17,18]. Most depsides and tridepsides are found in the lichen medulla. In contrast to cortical chemistry, which was usually correlated with higher taxonomic ranks, medullary chemistry was used as the species level discriminator [19]. However, in the last decades DNA techniques have changed the understanding of lichen taxonomy. As Lucking et al. explain, the presence of chemical compounds must be taken into account when comparing variations in well-established, molecularly defined taxa (e.g., chemosyndromes) [20]. Polyketides synthases (PKSs) genes for depside synthesis are currently being studied. Through the study of *Pseudoevernia furfuracea* chemotypes, Singh et al. have identified that the same biosynthetic gene cluster can produce different compounds, suggesting intraspecific variation in the regulation of metabolite synthesis [21].

The interest in depsides and tridepsides has grown in recent years due to their biological and pharmacological activities, as is reflected in the exponential increase in scientific publications. These compounds have shown antioxidant, cytotoxic, antimicrobial, anti-inflammatory, analgesic, and UV-protectant activities, among others [22,23,24,25,26].

This review aims to investigate the research findings related to the biological effects and mechanisms of action of lichen depsides and tridepsides in order to highlight the strong demand for an in-depth study of their structures and activities, ADMET parameter properties, and toxicities for the further development of drugs with lichen metabolites.

## 2. Pharmacological Activity of Lichen Depsides and Tridepsides

### 2.1. Depsides

**Figure 1:**
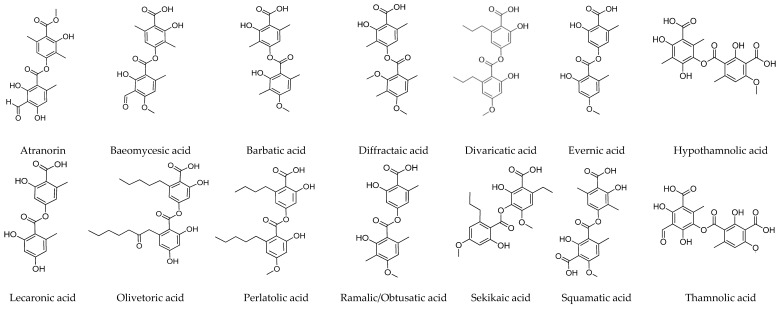
Chemical structures of different depsides of lichens.

The depside chemical structures are shown in Figure 1. The pharmacological activities of the depsides are compiled in Table 1.

#### 2.1.1. Atranorin

Atranorin is the most studied depside of this group of compounds. Several activities were investigated in in vitro, in silico, and even in vivo experiments. This review describes the activities, which range from the analgesic, anti-inflammatory, antiulcer, antidiabetic, antioxidant, and cytotoxic to the antimicrobial, antifungal, antiviral, antiparasitic, and larvicidal, as well as the potential neuroprotection activities, among others.

Different studies showed the effects of this depside against bacteria, fungi, viruses, protozoans, and larvae. Atranorin exhibited low antimicrobial activity against Gram-positive and Gram-negative bacteria, with high minimum inhibitory concentration (MIC) values (15.6 µg to 500 µg per disk against 10^7^ cells) [27]. Other studies showed the bacteriostatic effect against Methicillin-susceptible *Staphylococcus aureus* (MSSA) and Methicillin-resistant *Staphylococcus aureus* (MRSA) strains, isolated from cystic fibrosis patients, with differences among the strains (the MIC values for all the strains tested were 128 μg/mL, except that from MRSA Sa15, which was 64 μg/mL). Moreover, this depside was found to be effective against the adhesion of *Staphylococcus aureus* strains on polystyrene, reducing the ability of biofilm formation; it was also interesting with regard to the eradication of preformed biofilms [28]. Fungal species such as *Candida albicans* were minimally inhibited by atranorin [27,29]. Conversely, this compound showed its maximum antifungal activity against *Sclerotium rolfsii* (ED_50_ 39.70 µg/mL) [30]. In vitro activity against species of the *Mycobacterium* genera, such as *M. tuberculosis*, *M. aurum*, and diverse mycobacterial multidrug-resistant strains (MDR-A8, MDR-V791, MDR-R, MDR-40s), was also low, with MIC values of 250 μg/mL, 125 µg/mL, and 200 µg/mL, respectively [31,32,33]. Probiotic bacteria *Lactobacillus casei* increased its biomass (41.1 mg) due to the moderate growth stimulating activity of atranorin [34].

It also acts as an antiviral agent against the hepatitis C virus (HCV), inhibiting viral entry (IC_50_ 22.3 µM) [35].

As evidenced in the work of Zofou et al., atranorin also presented antiplasmodial effects against different strains and field isolates of *Plasmodium falciparum*, with IC_50_ values of <5 μM. Furthermore, this compound had synergistic interactions with artemether [36,37]. Other protozoa, such as piroplasm parasites (*Babesia bovis*, *Babesia bigemina*, *Babesia divergens*, *Babesia caballi* and *Theileria equi*), were affected by atranorin properties, reducing their multiplication in in vitro studies. Moreover, in vivo experiments were carried out using female BALB/c mice infected with *Babesia microti*. A reduction in *Babesia microti* multiplication by 68.17% was observed [38].

Larvicidal activity against the second and third instar larvae of the mosquito *Culiseta longiareolata* was also investigated, showing lethal concentration (LC) (50) and LC (90) values of 0.52 ppm and 5.93 ppm, respectively [39].

Several in vitro and in vivo studies demonstrated the analgesic and anti-inflammatory activities of atranorin. Due to the structure similarity between this depside and cyclooxygenase (COX) inhibitors, in vitro experiments were conducted, which showed 50% COX-1 inhibition at 17 μg/mL and 40% COX-2 inhibition at all concentrations of the analyzed range (17 μg/mL–0.17 μg/mL) [40]. In addition, according to Kumar and Müller, this compound inhibited leukotriene B4 (LTB4) biosynthesis in polymorphonuclear leukocytes (IC_50_ 6 µM) via a nonspecific redox mechanism [41]. Atranorin also exerted analgesic and anti-inflammatory effects in mice. With doses of 200 and 400 mg/kg (p.o.), a reduction (52.6 and 61.3%, respectively) in abdominal writhing and inhibition of the inflammatory processes was observed [42]. Furthermore, atranorin pretreatments (100, 200, and 400 mg/kg, i.p) reduced the effects on formalin- and capsaicin-induced orofacial pain tests, decreasing the nociceptive behavior of rubbing the face. At high doses (400 mg/kg, i.p), the effect appears to be central and is reversed with the administration of naxolone. Thus, in the acute model of carrageenan- and arachidonic acid-induced inflammation, atranorin reduced the formation of paw edema in rats [43].

In in vitro and in silico studies of N-substituted hydrazide derivatives of atranorin, a high α-glucosidase inhibition (IC_50_ 6.67 μM) was shown, making it a potential antidiabetic drug [44]. Atranorin showed high urease inhibition (IC_50_ 18.2 µM), making it a potential antiulcer drug candidate. Nonetheless, atranorin has no effectiveness against α-chymotrypsin [45].

Atranorin acts as an antioxidant or a prooxidant, depending on the study. Atranorin showed moderate antioxidant activity, as was evidenced in a 2,2-Diphenyl-1-picrylhydrazyl (DPPH) assay (IC_50_ value = 100 µg/mL), and anti-linoleic acid peroxidation activity (IC_50_ value = 116 µg/mL) and hydroxyl radical-scavenging activity (11.85 mg expressed as Trolox^®^ equivalents) in a total peroxyl radical-trapping (TRAP) assay (at 1–100 μg/mL concentrations) and a total antioxidant response (TAR) assay (at 100 μg/mL) [34,46,47]. Other studies presented atranorin as a weak antioxidant, as revealed in a beta-carotene-linoleate model system (14% antioxidant activity at 200 µg/mL) [48].

Conversely, a TBARS assay showed that atranorin induced lipoperoxidation at 0.1 to 100 μg/mL, increased NO production (only at high concentrations), and enhanced H_2_O_2_ formation. This compound acted as a superoxide scavenger, and hydroxyl radical/nitric oxide scavenging activity was not observed [47].

Cytotoxic effects on different cell lines (cancer and normal) were also investigated. Atranorin has demonstrated moderate activity against breast cancer cells such as MDA MB-231 and MCF-7, with IC_50_ values of 5.36 μM and 7.55 μM, respectively, by the downregulation of the Bcl-2, Akt, Bcl-w, and Bcl-xL proteins and the induction of Bax and caspase-3 expression. In silico studies confirmed the high interaction between the depside and the oncoproteins [49]. Cytotoxicity was also observed in human lung cancer cell lines (A549), epithelial carcinoma cell lines (SKHep1 and Huh-7), primary cancer cell lines (SNU-182), melanoma cell lines (HTB-140), prostate cancer cell lines (DU-145 and PC-3), and murine leukemia cell lines (P388) [50,51,52,53]. The tumorigenesis reduction and antimigratory activity against human lung cancer was mediated by the downregulation of activator protein 1 (AP-1), Wnt, and signal transducer and activator of transcription (STAT) pathways, as well as the inhibition of RhoGTPase activity [50]. Moreover, atranorin showed effects against hepatocellular carcinoma tumorigenesis by reducing cell proliferation (at 80 µg/mL), attenuating the cell cycle (G2/M phase cell cycle arrest), inducing cell death through necrosis, and diminishing metastatic potential by the suppression of cell migration and invasion [51]. Additionally, atranorin was found to be effective in inhibiting the cancer cell proliferation, migration, and actin cytoskeleton organization in melanoma cell lines (HTB-140) and prostate cancer lines (DU-145 and PC-3) [52]. The killing effect of atranorin on gastric cancer was also studied using complexes formed by superparamagnetic iron oxide nanoparticles (SPION) and atranorin. In vitro results on gastric cancer stem cells showed a reduction in proliferation, invasion, and tumorigenicity by reducing the expression of members of the Xc-/GPX4 axis and their mRNA 5-hydroxymethylcytidine modification and by inducing ferroptosis [54].

Moreover, this compound revealed cytotoxicity against all the cell lines (A2780, HCT-116 p53+/+ and HCT-116 p53−/−, SK-BR-3, HL-60, HT-29, Jurkat, and MCF-7) except HeLa, highlighting its activity against HL-60 cells. The clonogenic ability for the inhibition of all the tested tumor cells and the effects on the cell cycle at 200 µM (accumulation in S-phase at expense of G1/G0-phase) was also observed. In addition, atranorin seems to be effective as a pro-apoptotic agent with a p53-dependent action [55]. In particular, the studies on A2780 cancer cells and HT-19 also showed that atranorin caused cell death by reactive oxygen species/reactive nitrogen species (ROS/RNS) overproduction, caspase-3 activation, phosphatidylserine externalization, and mitochondrial membrane potential loss [56].

In vivo experiments were also conducted. Atranorin administration to BALB/c mice with T1-induced cancer disease was related to longer survival time, reduced tumor size, and higher numbers of apoptotic 4T1 cells. Comparing the effects of atranorin on normal mammary epithelial NMuMG cells and 4T1 cancer cells, it was observed that 4T1 cells were more sensitive to atranorin, reducing the clonogenic ability of carcinoma (75 µM), inducing apoptosis mediated by caspase-3 activation and poly ADP ribose polymerase (PARP) cleavage, and enhancing the depletion of Bcl-xL protein [57]. Moreover, atranorin SPION complexes showed tumorgenicity reduction in NOD-scid mice [54].

However, this depside showed low activity (IC_50_ ≥ 250 µg/mL) against melanoma cancer cell lines (A375, UACC-62, and B16-F10), human acute monocytic leukemia cell line (THP-1) (IC_50_ = 286 µg/mL), and human prostate cancer cells (LNCaP and DU-145), only inhibiting at the highest concentration tested (25 and 50 μM) [33,58,59,60]. Furthermore, atranorin was found to be ineffective as a topoisomerase I inhibitor [61]. Finally, in human lymphocytes, atranorin did not cause clastogenic and antiproliferative effects [16].

Atranorin participates in processes such as neuroprotection, acting as a neurotrophic and proneurogenic agent (131.73 μm in neurite outgrowth at 5 μM). Moreover, this compound stimulated neurotrophic genes (brain-derived neurotrophic factor (BDNF) and nerve growth factor (NGF) expression) [62].

Previous studies focused on the effects of atranorin alongside irradiation (360–366 nm), as evidenced in the inhibition of 8-methoxypsoralen (MOP)-human serum albumin (HSA), photobinding by 20.1%, and also as revealed in the significant hemolysis in red cell suspension; it acted as a photoprotective and photohemolytic agent, respectively [63,64].

#### 2.1.2. Baeomycesic Acid

The β-orcinol depside baeomycesic acid, a major secondary metabolite in *Thamnolia* spp., was shown to selectively inhibit 5-lipoxygenase (LOX); this seems to be related to its aromatic ring substitution pattern in positions 4, 1′ and 2′ [15,65]. However, this compound demonstrated weak activity against 12(S)-lipoxygenase in human platelets [66]. Moreover, this depside presented a slightly antiproliferative potential against cancer cell lines from several tissues (pancreas, ovary, and colorectal) [65].

#### 2.1.3. Barbatic Acid

Barbatic acid showed antiparasitic activity against the adult worms and larval stages of *Schistosoma mansoni* by causing death (IC_50_ value of 99.43 μM), affecting mobility, as was reflected in movements being presented only in the extremities, and triggering tegumentary damage [18,67]. Moreover, this depside was active against *Staphylococcus aureus* and *Enterococcus faecalis* (both the commercial and the clinic strains), with MIC values of 7.8 to 31.3 µg/mL [68]. This compound also displayed molluscicidal activity against *Biomphalaria glabrata* at 20 and 25 µg/mL [67].

Furthermore, this secondary metabolite was a potent cytotoxic agent against the lung cancer A549 cell line, with an IC_50_ value of 1.78 µM, by inducing apoptosis, as shown in the cell cycle arrest in the G0/G1 phase, the cleavage of PARP, and the activation of caspase-3 activity [69]. Conversely, this compound was found to have little effect in inhibiting the tumor promoter-induced Epstein–Barr virus (EBV), with an IC_50_ value higher than 100 µM [70]. Finally, in silico studies revealed that barbatic acid is a weak diuretic agent on the active site of the with-no-lysine kinase 1 (WNK1) domain [71].

#### 2.1.4. Diffractaic Acid

The antimicrobial activities of diffractaic acid were studied against bacteria and fungi. The inhibition zones in the *Staphylococcus aureus* and *Escherichia coli* cultures were 17.25 mm and 12.75 mm, respectively, at concentrations of 1000 ppm, exhibiting a strong activity (less than the amoxicillin control) [72]. This compound exhibited strong activity against *Fusarium fujikuroi* (MIC value of 16 × 10^−3^ mg/mL). This activity was higher than that of the flucytosine, clotrimazole, and ketoconazole drugs and similar to that of amphotericin B and posaconazole [73]. Furthermore, diffractaic acid was a potent antimycobacterial agent, with an MIC value of 15.6 μg/mL [31].

Diffractaic acid has also been investigated for its analgesic, antipyretic, and anti-inflammatory properties. This compound, at a dose of 200 mg/kg, exerted a moderate analgesic effect and a hypothermic effect on normal body temperature in male ddY mice. However, diffractaic acid did not suppress the fever in mice with lipopolysaccharide(LPS)-induced hyperthermia [14]. Moreover, diffractaic acid inhibited the formation of LTB4 in polymorphonuclear leukocytes, with an IC_50_ value of 8 µM via a nonspecific redox mechanism [41].

This secondary metabolite showed a protective effect against indomethacin-induced gastric ulcers in Wistar rats via increasing the antioxidant capacity (augmented enzyme activities and reduced glutathione levels and decreased lipid peroxidation) and via suppressing neutrophil infiltration. As an index of the neutrophil infiltration, myeloperoxidase (MPx), and nitric oxide synthase (NOS) activities were used. In gastric mucosal lesions, the activities of MPx and inducible nitric oxide synthase (iNOS) were increased. Diffractaic acid reduced MPx and iNOS activities and increased constitutive nitric oxide synthase (cNOS) activity [74].

Several works have focused on studying the cytotoxic activity of diffractaic acid in different types of tumor cells. This secondary metabolite showed moderate cytotoxicity in the cells of the nervous system (IC_50_ values of 122.26 mg/L in neurons and IC_50_ values of 35.67 mg/L in glioblastoma multiforme cells) [75]. Moreover, this depside exhibited a cytostatic effect through antiproliferation activity in the human keratinocyte HaCaT cell line, with an IC_50_ value of 2.6 mM [76]. Furthermore, diffractaic acid demonstrated a significant cytotoxicity against human breast cancer (MCF-7 cell line), human epithelial carcinoma (HeLa cell line), and human lung cancer (NCI-H460 cell line) at a concentration of 100 µg/mL [77]. In another study, this lichen compound displayed a strong proliferative action against the colon carcinoma HCT-116 cell line (IC_50_ value of 42.2 μM) and moderate activity against the breast adenocarcinoma MCF-7 cell line and the cervix adenocarcinoma HeLa cell line (IC_50_ values of 93.4 μM and 64.6 μM, respectively) [78]. In addition, diffractaic acid was found to be a moderate inhibitor of thioredoxin reductase [79]. Likewise, this compound was found to have little effect in inhibiting the tumor promoter-induced Epstein–Barr virus (IC_50_ value of >100 µM) [70].

All these studied activities led the investigation of new formulations that reduce the cytotoxicity of the treatment with diffractaic acid. The encapsulation of the compound with 2-hydroxypropyl-B-cyclodextrin on PLC microspheres improved its solubility and reduced the cytotoxicity in monkey kidney fibroblasts (Vero cells) [80].

#### 2.1.5. Divaricatic Acid

Divaricatic acid has resulted in being an effective antimicrobial agent against Gram-positive bacteria, with MIC values ranging from 7.0 μg/mL for *Bacillus subtilis* to 64.0 μg/mL for *Staphylococcus aureus*, highlighting its therapeutic role in methicillin-resistant *Staphylococcus aureus* (MRSA) infections. Moreover, this compound showed anti-*Candida* activity (MIC value of 20 μg/mL) [81]. Divaricatic acid, which is the major compound of the ether extract of the lichen *Ramalina aspera*, showed molluscicidal activities against *Biomphalaria glabrata* and cercaricidal activities against *Schistosoma mansoni* [82]. In another study, Silva et al. also demonstrated the antiparasitic properties of divaricatic acid against *Schistosoma mansoni* worms by affecting motility and viability (IC_50_ 100.6 μM). Indeed, this compound resulted in being not cytotoxic against human peripheral blood mononuclear cells, suggesting that it is safe for humans [83].

Cytotoxic activities were also investigated in UACC-62 human and B16-F10 murine melanoma cancer cells and NIH/3T3 fibroblasts using a sulforhodamine B assay. This depside exhibited strong activity against UACC-62 (GI_50_ 7µM) and B16-F10 cells (GI_50_ 11.3 µM), being more selective against melanoma cells than 3T3 normal cells (GI_50_ 37.3 µM) [59].

#### 2.1.6. Evernic Acid

Evernic acid acts as an antimicrobial agent, inhibiting the growth of *Staphylococcus aureus*, *Escherichia coli*, *Pseudomonas aeruginosa*, and *Candida albicans* (MIC values from 0.98 to 125 µg/mL) [84]. In deepening the mechanism of these effects, it was observed that the virulence of the Gram-negative opportunistic pathogen *Pseudomonas aeruginosa* was reduced by inhibiting quorum sensing on diverse *Pseudomonas aeruginosa* strains (54% of gfp expression of *lasB-gfp* and 50% of *rhlA-gfp* at a concentration of 116 µM). These genes are essential in the process because they encode the virulence factors elastase and rhamnolipids [23]. In another study, this depside diminished the maturation and growth of *Candida albicans* biofilms (Minimal Biofilm Inhibition Concentration (MBIC_50_) ≤ 12.5 µg/mL)) [85]. Moreover, this lichen compound displayed activity against the liver stage of the malaria parasite *Plasmodium*, targeting the fatty acid synthesis (FAS)-II pathway [86].

In another study, Férnandez-Moriano et al. investigated the neuroprotective activity of evernic acid, based on its antioxidant properties, in a model of oxidative stress, which was hydrogen peroxide-induced in astrocytes and neurons. This compound increased cell viability and reduced/oxidized the glutathione (GSH/GSSG) ratio and antioxidant enzymes expression. Moreover, evernic acid reduced lipid peroxidation, intracellular ROS overproduction, protein carbonyls content, and caspase-3 activity. The activation of the nuclear factor erythroid 2–related factor 2 (Nrf2) pathway contributes to this neuroprotection [87]. Neuroprotective effects were also shown in an MPTP-induced Parkinson’s disease model. Hence, evernic acid inhibited apoptosis and mitochondrial dysfunction, and it reduced oxidative stress in primary neurons. Moreover, a reduction was demonstrated in motor dysfunction and in dopaminergic neuronal death and astroglia activation using a C57BL/6 mouse model [88].

In terms of cytotoxic activities, evernic acid showed low cytotoxicity for the malignant mesothelioma cell line (MM98), the vulvar carcinoma cell line (A431), and the human keratinocyte cell line (HaCaT). There was also no effect on the stimulation of cell migration as measured by scratch healing assays in HaCaT [89]. Contrary to these data, evernic acid demonstrated strong cytotoxic activity at 25 and 50 µg/mL concentrations in a HeLa cell line and a reduction in A549 cancer cell proliferation (at 12.5, 25, 50, and 100 µg/mL). These depside concentrations were studied in healthy HUVEC cells with no toxic results, making this a good candidate for cancer treatment [90,91]. Evernic acid was also investigated against glioblastoma multiforme (GBM) cancer using A-172 and T98G cells, with moderate activity in A-172 cultures (IC_50_ 33.2 µg/mL). Multiple targets play a significant role in brain tumors, such as an immunosuppressive environment, inflammation, the degradation of hyaluronic acid, oxidative stress, and acetylcholine cholinesterase. Evernic acid also showed an inhibitory activity against indoleamine-2,3 dioxygenase 1 (IDO1), COX-2, hyaluronidase, and butyrylcholinesterase [92]. It should be also noted that evernic acid is a moderate inhibitor of tumor promoter-induced Epstein–Barr virus activation (64.6% of an inhibitory effect at a concentration of 50 µM) [71]. Finally, regarding the toxicity of this compound, in silico prediction tests showed no mutagenic effects, no tumorigenic effects, no reproductive alterations, and no irritant effects [84].

#### 2.1.7. Isolecanoric Acid

Isolecanoric acid has shown a prolonged antioxidant action. Based on the antioxidant properties, de Pedro et al. investigated its protective role in two neurodegenerative diseases models (L-BMAA for the Amyotrophic lateral sclerosis model and rotenone for the Parkinson’s disease model) in the human dopaminergic neuroblastoma SH-SY5Y cell line. Pretreatments with 10 and 25 µM of isolecanoric acid prevented mitochondrial dysfunction by decreasing the mitochondrial membrane potential (ΔΨm), reduced oxidative stress by attenuating the ROS production, attenuated early and late apoptosis, and inhibited glycogen synthase kinase-3 beta (GSK3β) and casein kinase I (CK1) [93].

#### 2.1.8. Lecanoric Acid

Lecanoric acid is of interest as an antimicrobial and antihelmintic agent since it inhibits 100% of Gram-negative bacteria *Aliivibrio fischeri*, and it causes 80% mortality in nematode *Caenorhabditis elegans* at 100 µg/mL [4]. Moreover, lecanoric acid was active as an antimicrobial agent against a wide variety of bacteria and fungi, with MIC values of 0.5 to 1 mg/mL [24].

The antioxidant properties of this compound are controversial according to the studies. Therefore, according to Ristic et al., lecanoric acid showed a slight antioxidant capacity, as evidenced in the DPPH assay (IC_50_ value of 424.5 μg/mL) and the reducing power assay (0.0165 to 125 μg/mL) [24]. Jayaprakasha et al. also showed that this compound had weak–moderate antioxidant activity (36% antioxidant activity at 500 µg/mL using the beta-carotene-linoleate model system) [48]. However, according to Thadhani et al., lecanoric acid has good antioxidant activity compared to other lichen substances [superoxide radical (SOR) test (IC_50_ value of 91.5 µmol), DPPH (IC_50_ value of 34 µmol), and nitric oxide radical (NOR) test (IC50 value of 53.5 µmol)] [94].

Studies on the cytotoxic activity of lecanoric acid showed that this depside has moderate activity against colon HCT116 cancer cells and reduced cell colony formation by decreasing Axin2 expression and M phase arrest (downregulation of CDK1, upregulation of cyclinB1 and pH3) [95,96]. Lecanoric acid also exhibited slight activity against human larynx carcinoma Hep-2 cells, human breast carcinoma MCF7 cells, human kidney carcinoma 786-0 cells, murine melanoma B16-F10 cells (IC_50_ values > 50 µg/mL), Hela cells (IC_50_ value of 124 μg/mL), and A549 cells and LS174 cells (IC_50_ value of 200 μg/mL) [24,97]. Moreover, lecanoric acid was an effective thioredoxin reductase inhibitor for cancer therapy, even more effective than the common antitumoral drugs such as doxorubicin and cisplatin [79].

Finally, in vitro studies revealed that lecanoric acid exhibited effective α-glucosidase inhibitory activity (85.9% of inhibition) with an IC_50_ value of 350 µM [98] and moderate protein tyrosine phosphatase 1B (PTP1B) inhibitory activity (IC_50_ 31 μM) [99].

#### 2.1.9. Methyl Evernate

Methyl evernate has displayed antimicrobial activity against bacteria and fungi and is especially active against *Bacillus cereus* (MIC value of 0.125 mg/mL) and *Candida albicans* (MIC value of 0.25 mg/mL). Furthermore, methyl evernate had low DPPH radical scavenging activity (IC_50_ value of 391.57 μg/mL) and higher reducing power than acetone extracts of *Ramalina* spp. [24]. Moreover, this depside inhibited the cancer cell growth of the human epithelial carcinoma Hela cell line (IC_50_ value of 46.45 μg/mL), the human lung carcinoma A549 cell line (IC_50_ value of 76.84 μg/mL), and the human colon carcinoma LS174 cell line (IC_50_ value of 161.37 μg/mL).

#### 2.1.10. Olivetoric Acid

Olivetoric acid has antimicrobial activity against a wide range of bacteria, yeast, and fungi. This compound was active against 12 of 15 species of bacteria and yeast (it was inactive in *Klebsiella pneumoniae*, *Pseudomonas aeruginosa*, and *Pseudomonas syringae*) and against 7 of 11 species of fungi (it was inactive against *Alternaria citri*, *Alternaria tenuissima*, *Aspergillus niger*, and *Gaeumannomyces graminis*) [100].

Olivetoric acid showed slight to moderate antioxidant properties in cultured human amnion fibroblasts (total antioxidant capacity value of 20.79 mmol Trolox equivalent/L) and in cultured human lymphocytes (HLs) (total antioxidant capacity value of 3.79 mmol Trolox equivalent/L) [16,101]. Finally, olivetoric acid induced cytotoxicity and genotoxicity against the glioblastoma multiforme U87MG cell line (IC_50_ value of 17.55 mg/L) and primary rat cerebral cortex (PRCC) cells (IC_50_ value of 125.71 mg/mL) via oxidative stress-induction, as evidenced by the lactate dehydrogenase (LDH) activity and oxidative DNA damage [102]. Moreover, concentrations of 100–400 mg/L of olivetoric acid showed activity against human hepatocellular carcinoma cells (HepG2) and the upregulation of the pro-apoptotic genes, BAK, CASP6, CASP7, CASP8, FADD, FAS, and FASLG [103]. Furthermore, this depside has resulted in being of interest as anti-angiogenic agent, as evidenced by its ability to prevent rat adipose tissue endothelial cell (RATECs) cell proliferation by disrupting microtubules and inhibiting actin polymerization [104].

#### 2.1.11. Perlatolic Acid

Perlatolic acid has good antimicrobial properties against methicillin-resistant *Staphylococcus aureus* strains, with an MIC_90_ value of 32 µg/mL, and it showed a synergic action with gentamicin and an antagonism action with levofloxacin [105].

This compounds also exerted neurobiological processes such as neuroprotection, neurotrophicity, and neurogenesis. Hence, this depside acts as a neurotrophic and proneurogenic agent (125.34 μm in neurite outgrowth at 0.5 μM) by inducing the upregulation of neurotrophic genes (BDNF and NGF). This neurotrophic activity is also related to the increased histone acetylation of H3 and H4 protein in a mouse neuroblastoma (Neuro2A) cell line. Moreover, this secondary metabolite was a potent acetylcholinesterase (AChE) inhibitor (IC_50_ 6.8 μM) [63]. Moreover, in silico, in vitro, and in vivo studies have shown that perlatolic acid is a potent anti-inflammatory compound by inhibiting microsomal prostaglandin E2 synthase-1 (IC_50_ 0.4 µM), 5-lipoxygenase (IC_50_ 1.8 µM for cell-based assay and IC_50_ 0.4 µM for purified enzyme), tumor necrosis factor alpha-induced nuclear factor kappa B (IC_50_ 7 µM), and leukocyte recruitment [106,107]. Furthermore, perlatolic acid showed slight to moderate immune-modulating properties in cultures of peritoneal macrophage cells from mice, as evidenced in a significant increase in hydrogen peroxide release and a slight increase in nitric oxide (NO) release activity [108].

#### 2.1.12. Ramalic Acid/Obtusatic Acid

Ramalic acid/Obtusatic acid was active as an antimicrobial agent against five bacteria and ten fungal species with MIC values from 0.125 to 1 mg/mL [24]. Moreover, this depside showed slight to moderate antioxidant activity (DPPH radical scavenging activity with IC_50_ value of 324.61 μg/mL and reducing power of 0.0142 at 125 µg/mL) [24]. Furthermore, ramalic acid/obtusatic acid showed weak to moderate cytotoxic activity against the human epithelial carcinoma (Hela) cell line, the human lung carcinoma (A549) cell line, and the human colon carcinoma (LS174) cell line with IC_50_ values of 43.24 μg/mL, 93.98 μg/mL, and 74.28 μg/mL [24]. On the other hand, this secondary metabolite was inactive as an inhibitor of LTB4 production via non-mediation by redox reactions and as an antiproliferative agent against the human keratinocyte HaCaT cell line [41,76].

#### 2.1.13. Sekikaic Acid

Sekikaic acid displayed maximum antimicrobial activity against *Escherichia coli* (78% inhibition), moderate activity against *Streptococcus mutans*, *Staphylococcus aureus*, and *Streptomyces viridochromogenes* (60%, 50%, and 55% inhibition, respectively), and low activity against *Bacillus subtilis* (15% inhibition) [109]. In addition, sekikaic acid acts as a potent antiviral agent against a recombinant strain rg respiratory syncytial virus (IC_50_ 5.69 µg/mL) and a respiratory syncytial virus A2 strain (IC_50_ 7.7 µg/mL) by interfering with viral replication at a viral post-entry step. Its antiviral action is even higher than the reference compound ribavirin [110]. Moreover, sekikaic acid has good antioxidant properties, as revealed by hydroxyl radical assay (IC_50_ value of 41.5 µg/mL), ferric ion assay (IC_50_ value of 42.0 µg/mL), DPPH assay (IC_50_ value of 11.24 µg/mL; IC_50_ value of 32.6 µmol), and SOR assay (IC_50_ value of 82.0 µmol); this is attributed to the three hydroxyl groups of its structure [94,109,111]. In vivo studies demonstrated that this depside has antidiabetic activity by inhibiting digestive enzymes (α-glucosidase and α-amylase) and by reducing plasma glucose levels (44.17%) in a streptozotocin-induced type 2 diabetic albino rat model [111]. Regarding its cytotoxicity activity, sekikaic acid was inactive against A2780 ovarian and MCF-7 breast cancer cell lines [112].

#### 2.1.14. Squamatic Acid

Squamatic acid showed weak antimicrobial activity against *Staphylococcus aureus* (MIC = 1250.0 mg/mL) [113]. Moreover, this compound displayed a weak antiproliferative effect on prostate cancer PC-3 cells (IG_50_ value of >200 µM) [114].

#### 2.1.15. Thamnolic Acid

Thamnolic acid showed antimicrobial activity against fungi (*Alternaria alternata*, *Aspergillus fumigatus*, and *Sclerotium rolfsii*, with MIC values of 400, 400, and 200 µg/mL, respectively), yeast (*Candida krusei*, with an MIC value of 400 µg/mL), and bacteria (*Bacillus cereus*, *Bacillus subtilis* and *Proteus vulgaris*, with an MIC value of 400 µg/mL, and *Listeria monocytogenes* and *Micrococcus luteus*, with an MIC value of 200 µg/mL) [115]. On the other hand, this depside showed weak antiproliferative activity in prostate cancer cells (IC_50_ value of >200 µM) [114].

**Table 1:** Pharmacological activity of lichen depsides.

Depside	Botanical Origin	Type of Study	Experimental Model	Activities	Results	References
Atranorin	*Parmotrema saccatilobum* (Taylor) Hale	In vitro	Cyclooxygenase inhibition assay	Analgesic	Inhibition of COX-1 (IC_50_ 45 μM). Inhibition (40%) of COX-2 ranging between 17 μg/mL and 0.17 μg/mL.	[40]
	*Cladina kalbi.* (Ahti)	In vivo	Male Swiss mice	Analgesic	Acetic acid-induced writhing test—200 and 400 mg/kg (p.o.)—reduction (*p* < 0.05) abdominal writhing by 52.6 and 61.3%, compared to control. Formalin test—200 and 400 mg/kg (p.o.) inhibition inflammatory processes (second phase) dose dependently.	[42]
	*Cladina kalbi.* (Ahti)	In vivo	Male Swiss mice	Analgesic Anti-inflammatory	Inhibitory effect in formalin- and capsaicin-induced orofacial pain tests. Anti-inflammatory effects in the acute model of inflammation (leukocyte migration to the peritoneal cavity), carrageenan- and arachidonic acid-induced paw edema in rats.	[43]
	*-*	In vitro In silico	α-Glucosidase assay HEK293 (Human embryonic kidney cell line) Docking studies	Antidiabetic	N-substituted hydrazide derivatives of atranorin, more potent inhibition than the original. Weak or no cytotoxicity toward HEK293 cell line.	[44]
	*Parmelia nepalensis* (Taylor)	In vitro	Polymorphonuclear leukocytes	Anti-inflammatory	Inhibition of LTB4 biosynthesis via non-redox mechanism.	[41]
Atranorin	*Kigelia africana* (Lam.) Benth	In vitro	Chloroquine-resistant W-2 and two field isolates (CAM10 and SHF4) of *Plasmodium falciparum* LLC/MK2 monkey kidney cells	Antimalarial	Good activity against all parasite strains (IC_50_ < 5 μM). Cytotoxicity at high concentrations.	[36]
	*Kigelia africana* (Lam.) Benth	In vitro	Multidrug-resistant W2mef strain of *Plasmodium falciparum*	Antimalarial	Parasite lactate dehydrogenase assay (IC_50_ 1.78 μM). Synergistic effects with artemether.	[37]
	*Homalia trichomanoides* (Hedw.) B. S. G.	In vitro	*Candida albicans*	Antimicrobial	Minimum inhibitory doses of 2.0 µg.	[29]
	*Parmelia reticulata* (Taylor)	In vitro	*Sclerotium rolfsii*, *Rhizoctonia solani*, *R. bataticola*, *Fusarium udum*, *Pythium aphanidermatum* and *Pythium debaryanum*	Antimicrobial	Maximum antifungal activity against *Sclerotium rolfsii* (ED_50_: 39.70 µg/mL).	[30]
	*Cladonia foliacea* (Huds.) Willd	In vitro	Gram-positive bacteria: *Bacillus cereus*, *Bacillus subtilis*, *Staphylococcus aureus*, *Streptococcus faecalis*, *Listeria monocytogenes* Gram-negative bacteria: *Proteus vulgaris*, *Aeromonas hydrophila.* Fungi: *Candida albicans*, *Candida glabrata*	Antimicrobial	Low activity with high MIC values (15.6 µg to 500 µg per disk against 10^7^ cells).	[27]
	*Parmotrema dilatatum* (Vain.) Hale, *Parmotrema tinctorum* (Nyl.) Hale	In vitro	*Mycobacterium tuberculosis*	Antimicrobial	Low-activity compound (MIC value 250 μg/mL).	[31]
Atranorin	*-*	In vitro	Methicillin-resistant *Staphylococcus aureus* strains	Antimicrobial	Effective in counteracting adhesion to polystyrene, against biofilm formation and against MRSA.	[28]
	*Stereocaulon alpinum* Laurer.	In vitro	*Mycobacterium aurum* strains	Antimicrobial	Low-activity MIC values >/= 125 µg/mL.	[32]
	*Usnea laevis* Nyl.	In vitro	*Mycobacterium tuberculosis* Mycobacterial multidrug-resistant (MDR) strains (MDR-A8, MDR-V791, MDR-R, MDR-40)	Antimicrobial	Inactive against mycobacterial strains MIC values ≥ 200 µg/mL.	[33]
	*Cladina kalbii* Ahti	In vitro	TRAP, TAR, TBARS, hydroxyl radical scavenging activity, nitric oxide scavenging activity, CAT- SOD-like activity. SH-SY5Y neuroblastoma cell line	Antioxidant	TRAP assay: 1–100 μg/mL significant antioxidant effects (dose-dependent). TAR assay: 100 μg/mL significant antioxidant capacity. TBARS: 0.1 to 100 μg/mL AAPH-induced lipoperoxidation. No hydroxyl radical/nitric oxide scavenging activity. Increase (↑) H_2_O_2_ formation in vitro ↑ superoxide degradation.	[47]
	*Parmotrema austrosinense* (Zahlbr.) Hale	In vitro	DPPH assay Anti-linoleic acid peroxidation activity	Antioxidant	IC_50_: 100 µg/mL. IC_50_: 116 µg/mL.	[34]
	*Hypotrachyna revoluta* (Flörke) Hale	In vitro	Hydroxyl radical-scavenging activity	Antioxidant	Metabolite (11.8 mg) same activity as Trolox (1 mg).	[46]
	*Parmotrema stuppeum* (Taylor) Hale	In vitro	Beta-carotene-linoleate model system	Antioxidant	14% of antioxidant activity at 200 µg/mL.	[48]
Atranorin	*-*	In vitro	Piroplasm parasites: *Babesia. bovis*, *Babesia bigemina*, *Babesia divergens*, *Babesia caballi*, and *Theileria equi* Hosts of piroplasm parasites: human foreskin fibroblasts (HFF), mouse embryonic fibroblast (NIH/3T3) Madin–Darby bovine kidney (MDBK)	Anti-parasitic	Suppression of multiplication: IC _50_ *(B. bovis*): 98.4 µM, IC_50_ (*B. bigemina*): 64.5 µM, IC_50_ (*B. divergens*): 45.2 µM, IC_50_ (*B. caballi*): 46.6 µM, IC_50_ (*T. equi*): 71.3 µM. Reduce (↓) Cell viability.	[38]
	*-*	In vivo	BALB/c mice infected by *B. microti*	Anti-parasitic	↓ *B. microti* multiplication in mice by 68.17%.	[38]
	*-*	In vivo	Normal mammary epithelial NMuMG cells BALB/c mice with T1-induced cancer disease	Antitumoral	↓ Clonogenic ability of carcinoma. ↑ Apoptosis associated with the activation of caspase-3 and PARP cleavage in 4T1 cells. ↑ Depletion of Bcl-xL protein in 4T1 cells. Longer survival time, reduced tumor size, and higher numbers of apoptotic 4T1 cells. Normal NMuMG cells are less sensitive to ATR.	[57]
	*Stereospermum acuminatissimum* K. Schum.	In vitro	Urease inhibition assay Chymotrypsin inhibition assay	Antiulcerogenic	Excellent urease inhibition IC_50_ (18.2 µM). No α-chymotrypsin inhibitory effect.	[45]
	*Stereocaulon evolutum* Graewe.	In vitro	HCV grown in Huh-7.5.1 human hepatic cell line	Antiviral	Interferes with the lifecycle of hepatitis C virus (HCV), inhibiting only viral entry (IC_50_: 22.3 µM).	[35]
	*Parmotrema rampoddense* (Nyl.) Hale	In silico In vitro	Docking studies with breast cancer oncoproteins MDA MB-231 and MCF-7 (breast cancer cell lines)	Cytotoxic	Molecular docking studies interaction: Akt > Bax, Bcl-xL and Bcl-2 > Bcl-w proteins. IC_50_ (MDA MB-231) = 5.36 μM; IC_50_ (MCF-7) = 7.55 μM.	[49]
Atranorin	*Everniastrum vexans* (Zahlbr. ex W.L. Culb. and C.F. Culb.)	In vitro	A549 (human lung cancer cell line)	Cytotoxic	↓ Lung cancer cell motility and tumorigenesis by affecting AP-1, Wnt, and STAT signaling and suppressing RhoGTPase activity.	[50]
	*Stereocaulon caespitosum* Redinger	In vitro	SKHep1 and Huh-7 (epithelial carcinoma cell line) SNU-182 (primary cancer cell line)	Cytotoxic	↓ Cell growth at 80 µg/mL in all cell lines Cell cycle attenuated. ↑ Cell death through necrosis. ↓ Metastatic potential by suppression of cell migration and invasion.	[51]
	*-*	In vitro	HTB-140 (melanoma cell line) DU-145 and PC-3 (prostate cancers) normal human skin fibroblasts PNT2 (prostate epithelial cell line)	Cytotoxic	↓ Cancer cell proliferation, migration, and actin cytoskeleton organization.	[52]
	*Hypogymnia physodes* (L.) Nyl	In vitro	Human lymphocytes- cytochalasin-B blocked micronucleus (CBMN) assay.	Cytotoxic	No significant clastogenic and antiproliferative effects on selected concentrations.	[16]
	*-*	In vitro	A2780 (human ovarian cancer cell line) HT-29 (human colon cancer cell line)	Cytotoxic	Loss in the mitochondrial membrane potential. ↑ caspase-3 activation (only in HT-29 cells) and phosphatidylserine externalization. ↑ ROS/RNS. ↑ PARP, p53, Bcl-2/Bcl-xL, Bax, p38, pp38.	[56]
Atranorin	*-*	In vitro	A2780 (human ovarian carcinoma) HCT-116 p53+/+ and HCT-116 p53−/− (human colon carcinoma) HeLa (human cervix adenocarcinoma) SK-BR-3 (human breast adenocarcinoma) HL-60 (human promyelocytic leukemia) HT-29 (human colon adenocarcinoma) Jurkat (human T cells lymphocyte leukemia) MCF-7 (human breast adenocarcinoma)	Cytotoxic	Cytotoxicity against all cell lines except against HeLa (especially effective against HL-60 cells (50 μM). Clonogenic inhibition ability of all tested tumor cells. Accumulation in S-phase at expense of G1/G0-phase. Lower incidence in p53-deficient cells.	[55]
Atranorin SPION	*-*	In vitro	GCSCs (gastric cancer stem cells)	Cytotoxic	Inhibition proliferation, invasion, angiogenesis, and tumorigenicity of CD44+/CD24+. ↑ Oxidative stress. ↑ Fe^2+^ accumulation/ferroptosis. Increase mRNA encoding apoptosis factors, COX-2 levels. Inhibition GCSC markers and GPX4, NCOA4.BRF2, CD98. Downregulation mRNA hm5C modification levels.	[54]
	*-*	In vivo	NOD-scid mice	Cytotoxic Antitumor	Smaller tumors in weight and volume. Inhibition GPX4 and SLC7A11.	[54]
Atranorin	*Bacidia stipata* I. M. Lamb.	In vitro	A375 (melanoma cancer cell line)	Cytotoxic	Low inhibition (only high concentrations)	[55]
	*Parmotrema dilatatum* (Vain.) Hale	In vitro	UACC-62 and B16-F10 (melanoma cells) 3T3 (normal cells)	Cytotoxic	IC_50_: 250 µg/mL. Low cytotoxic effects on all the cell lines.	[59]
	*Bacidia stipata* I. M. Lamb.	In vitro	Androgen-sensitive (LNCaP) and androgen-insensitive (DU-145) human prostate cancer cells.	Cytotoxic	Lower activity inhibiting cancer cells only at higher concentrations (25 and 50 μM).	[60]
	*Ramalina glaucescens* Kremp.	In vitro	P388 murine leukemia cell line	Cytotoxic	Moderate activity against (IC_50_ of >33 µM).	[53]
	*Usnea laevis Nyl.*	In vitro	Human acute monocytic leukemia cell line (THP-1)	Cytotoxic	IC_50_: 286.13 µg/mL. Low cytotoxic effects on macrophages.	[33]
	*-*	In vitro	Calf thymus DNA	DNA-interacting agents	ATR acts as effective DNA-interacting agent. No inhibitory effect on Topo isomerase I.	[61]
	*-*	In vitro	Second and third instar larvae of the mosquito *Culiseta longiareolata*	Larvicidal activity	LC (50) values: 0.52 ppm. LC (90) values: 5.93 ppm.	[39]
	*Usnea articulata* (L.) Hoffm.	In vitro Ex vivo	Neuro2A (mouse neuroblastoma) cell line Primary neural stem or progenitor cells	Neuroprotective	Neurotrophic activity (131.73 μm at 5 μM). Gene expression of BDNF and NGF modulation.	[62]
	*Umbilicaria antarctica* Frey and I. M. Lamb.	In vitro	Red cell suspension	Photohemolytic	Significant hemolysis in a red cell suspension after irradiation of atranorin with 366 nm light. Higher in presence of nitrogen.	[64]
	*Umbilicaria antarctica* Frey and I. M. Lamb.	In vitro	Inhibition of 8-MOP-human serum albumin (HSA) photobinding.	Photoprotective	Atranorin (10 mM) and irradiation (360 nm) inhibited photobinding to HSA by 20.1%.	[63]
Atranorin	*Parmotrema austrosinense* (Zahlbr.) Hale	In vitro	Bacterial strain *Lactobacillus casei*	Probiotic bacteria	Moderate growth stimulating activity in terms of increased dry matter of biomass (41.1 mg) of *L. casei*.	[34]
Baeomycesic acid	*Thamnolia subuliformis* (Ehrh.) W. Culb.	In vitro	Porcine leucocytes Sheep seminal vesicle microsomes	Cytotoxic	Potent 5-lipoxygenase inhibitor (IC_50_ = 8.3 µM). Inactive against COX.	[15]
	*Thamnolia vermicularis* (Sw.) Schaer.	In vitro	Human platelets	Cytotoxic	Weak 12(S)-LOX inhibitor (14.7 +/− 2.76%).	[66]
	*Thamnolia vermicularis* (Sw.) Schaer.	In vitro	AGS (stomach cancer cell line) Capan-1, Capan-2 and PANC-1 (pancreas cell lines) HL-60, K-562 and JURKAT (blood cancer cell lines) NCI-H1417 (lung cancer cell line) NIH: OVCAR-3 (ovary cancer line) PC-3 (prostate cancer cell line) T47-D (breast cancer line) WiDr (colorectal cancer cell line)	Cytotoxic	Slight anti-proliferative activity. Selective 5-LOX inhibitor.	[65]
Barbatic acid	*Cladonia borealis* Stenroos	In vitro	*Staphylococcus. aureus* NEWP0023 *Enterococcus. faecalis* (NEWP0012) *Escherichia. coli* (NEWP 0022)	Antimicrobial	MIC values: *S. aureus* (NEWP0023) = 31.3 µg/mL; *S. aureus (clinic) =* 31.3 µg/mL; *E. faecalis* (NEWP0012) = 7.8 µg/mL; *E. faecalis* (clinic) = 31.3 µg/mL; *E. coli* = nt.	[68]
	*Cladia longissima* (Sw.) Nyl.	In vitro	Adult worms of *Schistosoma mansoni*	Antiparasitic	Schistosomicidal effect (death, tegumentary damages, and changes in mobility).	[18]
	*Cladia longissima* (Sw.) Nyl.	In vitro	Adult mollusks of *Biomphalaria glabrata* Cercariae of *Schistosoma mansoni*	Antiparasitic Antimolluscal	Molluscicidal activity against *B. glabrata* at 20 and 25 µg/mL. Schistosomicidal effect against the parasite *S. mansoni* at the second larval stage (1 µg/mL after 60 min of exposure).	[67]
Barbatic acid	*Usnea longissima Ach.*	In vitro	A549 (lung cancer cell line)	Cytotoxic	Pro-apoptotic effect (G0/G1 accumulation and poly ADP-ribose polymerase cleavage).	[69]
	*Usnea longissima Ach*	In vitro	Tissue culture	Cytotoxic	Slight inhibitor of tumor promoter-induced Epstein–Barr virus (EBV) activation.	[70]
	*Pyrrosia petiolosa* (Christ) Ching.	In silico	With-no-lysine 1 (WNK1) kinase	Diuretic	Weak diuretic potential.	[71]
Diffractaic acid	*Usnea diffracta* Vain.	In vivo	Male ddY mice Lipopolysaccharide (LPS)-induced (hyperthermia model) Acetic acid-induced writhing and tail-pressure method (analgesic model)	Analgesic and antipyretic	Hypothermic effect (dose of 200 mg/kg) on normal body temperature. Analgesic effect (dose of 200 mg/kg).	[14]
	*Parmelia nepalensis* (Taylor) *Parmelia tinctorum* Despr. Ex Nyl	In vitro	Polymorphonuclear leukocytes	Anti-inflammatory	Inhibition of LTB4 biosynthesis by specific enzyme interaction.	[41]
	*Usnea blepharea* Motyka	In vitro	Gram-positive bacteria: *Staphylococcus aureus*, Gram-negative bacteria: *Escherichia coli*	Antimicrobial	Antibacterial. Strong inhibition at 750 and 1000 ppm concentration.	[72]
	*-*	In vitro	*Fusarium fujokuroi*	Antimicrobial	Antifungal. MIC 16 × 10^−3^ mg/mL. Similar to amphotericin B, isovuconzole, terbafine, voriconazole.	[73]
	*Usnea subcavata* (Motyka)	In vitro	*Mycobacterium tuberculosis*	Antimicrobial	Anti-tubercular activity. High active compound (MIC value 15.6 μg/mL).	[31]
Diffractaic acid	*Usnea longissima* Ach	In vivo	Albino Wistar rats Indomethacin-induced gastric lesions	Antiulcerogenic	Significant gastroprotective effect. ↑ SOD and GPx activities and GSH levels ↓ lipid peroxidation ↓ myeloperoxidase and inducible NOS (iNOS) activities ↑ constitutive NOS (cNOS) activity.	[74]
	-	In vitro	U87MG (glioblastoma multiforme cell line) PRCC cells (neurons from Sprague Dawley^®^ rats)	Cytotoxic	IC_50_ value (PRCC) = 122.26 mg/L. IC_50_ value (U87MG) = 35.67 mg/L. High antioxidant capacity on PRCC cells (10 mg/L).	[75]
	*Parmelia nepalensis* (Taylor) *Parmelia tinctorum* Despr. ex Nyl	In vitro	HaCaT (human keratinocyte cell line)	Cytotoxic	Inhibition cell growth (IC_50_ values of 2.6 mM). No changes on LDH activity, cytostatic effects.	[76]
	*Usnea aciculifera* Vain.	In vitro	HeLa (human epithelial carcinoma cell line) NCI-H460 (human lung cancer cell line) MCF-7 (human breast cancer cell line)	Cytotoxic	Strong cytotoxic activity against all cell lines (100 μg/mL).	[77]
	*Protousnea magellanica* (Mont.) Krog	In vitro	MCF-7 (breast adenocarcinoma cell line) HeLa (cervix adenocarcinoma cell line) HCT-116 (colon carcinoma cell line)	Cytotoxic	Cytotoxic effects in a concentration-dependent manner (2.5–100 μM). No increase intracellular ROS level. No prevention of oxidative injury induced by t-butylhydroperoxide in HeLa cells.	[78]
Diffractaic acid	*-*	In vitro	Mitochondrial TrxR purified from rat lung	Cytotoxic	Moderate inhibitory effect on Thioredoxin reductase (TrxR).	[79]
	*Usnea longissima* Ach	In vitro	Tissue culture	Cytotoxic	Slight inhibitor of tumor promoter-induced Epstein–Barr virus (EBV) activation.	[14]
	*Usnea longissima* Ach	In vivo	Titanium-implanted rabbits	Proapoptotic agent	↑ Caspase-2, Csp-8, Csp-9, and Csp-3 activation. ↑ Strong myeloperoxidase and inducible nitric oxide synthase activities. ↓ SOD activity and total glutathione level.	[116]
Divaricatic acid	*Evernia mesomorpha Nyl.*	In vitro	Gram-positive bacteria: *Staphylococcus aureus*, *Enterococcus faecium*, *Bacillus subtilis, Micrococcus luteus*, *Streptococcus epidermidis*, *Streptococcus mutans* Gram-negative bacteria: *Escherichia coli*, *Pseudomonas aeruginosa*, *Klebsiella pneumoniae*, *Salmonella typhimurium*, *Vibrio vulnificus* Fungi: *Candida albicans*	Antimicrobial	Effective against Gram+ bacteria (MIC values ranging from 7.0 to 64.0 μg/mL) and *Candida albicans.*	[81]
	*-*	In vitro	Gram-positive bacteria: *Staphylococcus aureus* Gram-negative bacteria: *Escherichia coli*, Mycobacteria: *Mycobacterium tuberculosis* Protozoan: *Plasmodium berghei* liver stage (LS) parasites, *Plasmodium falciparum* blood stage (BS) parasites	Antimicrobial Antiplasmodial	No antibacterial/antimycobacterial activity. Low antiplasmodial activity. Low LS activity (IC_50_ = 77.3 μM), high BS potential (IC_50_ = 142.1 μM). Plasmodial FAS-II enzyme (*PfFabI* and *PfFabZ*) inhibition.	[86]
Divaricatic acid	*-*	In vitro	*Pseudomonas aeruginosa*	Antimicrobial	↓ *Pseudomonas aeruginosa* virulence factors expression by inhibiting quorum sensing.	[23]
	*Ramalina aspera* Räsänen	In vitro	Mollusk *Biomphalaria glabrata* Cercariae of the helminth *Schistosoma mansoni*	Molluscicidal and cercaricide	High toxicity against: adult snails (5 μg/mL) and embryos (20 μg/mL after 6 h of exposure) cercariae (10 μg/mL after 30 min of exposure).	[82]
	*Dirinaria aspera* Hasanen	In vitro	UACC-62 and B16-F10 (human and murine melanoma cells) 3T3 normal cells	Cytotoxic	Cytotoxic against both lines (LC_50_ 50.2 μM (UACC-62) LC_50_ 643.7 Μm (B16-F10). More selective against melanoma cells than normal cells.	[59]
	*Canoparmelia texana*	In vitro	PBMCs (peripheral blood mononuclear cell)	Cytotoxic	No cytotoxicity (IC_50_ > 200 μM).	[82]
	*Cetraria ornata Müll.Arg.*	In vitro	Tissue culture	Cytotoxic	Moderate inhibitor of tumor promoter-induced Epstein–Barr virus (EBV) activation.	[70]
Evernic acid	*Evernia prunastri* (L.) Ach.	In vitro	Gram-positive bacteria: *Staphylococcus aureus* Gram-negative bacteria: *Pseudomonas aeruginosa*, *Escherichia coli* Fungi: *Candida albicans*	Antimicrobial	Inhibition of the growth of all tested microorganisms (MIC values = from 0.98 to 125 µg/mL).	[84]
	*Evernia prunastri* (L.) Ach.	In silico	Prediction of toxicity risk based on fragment-based toxicity estimation	Toxicity	No mutagenic, no tumorigenic, no reproductive alterations and no irritant effects.	[84]
	*Evernia prunastri* (L.) Ach.	In vitro	*Candida albicans* biofilms	Antimicrobial	Slow maturation and reduction in biofilms with MBIC_50_ ≤ 12.5 µg/mL.	[85]
	*Evernia prunastri* (L.) Ach.	In vitro	HeLa (Human epithelial cervical cancer)	Cytotoxic	Strong cytotoxic and antiproliferative effects (25 and 50 µg/mL).	[91]
Evernic acid	*Evernia prunastri* (L.) *Pseudoevernia furfuraceae* (L.) Zopf.	In vitro	A549 (human lung cancer cells) HUVEC (umbilical vein endothelial cells)	Cytotoxic	No significant effects in healthy cells. Decrease in proliferation in cancer cells (12.5–100 µg/mL).	[90]
	*Evernia prunastri* (L.) Ach.	In vitro	Glioblastoma multiforme cell line: A-172 and T98G cell lines.	Cytotoxic	Reduction A-172 cell viability at 10 µM. Mildly cytotoxic on T98G cell line. Anti-IDO1 (32.8 % inhibition). Anti-COX-2 (50.7%) inhibition. Anti-hyaluronidase activity (IC_50_ 600 µg/mL). Weak antioxidant properties (DPPH (750 µg/mL) CUPRAC (250 µg/mL)) (21.2 % SOD and 20 % GPx inhibition). Inhibition of BChE. (85.9 %) No AchE inhibition. BBB Permeability (8.6 × 10^−6^ (cm/s) at 4 h.	[92]
	*Evernia prunastri* (L.) Ach.	In vitro	U373-MG (human glioblastoma astrocytoma cell line) SH-SY5Y (human neuroblastoma cell line)	Neuroprotective	↑ Cell viability; GSH/GSSG ratio; antioxidant enzymes expression. ↓ ROS; lipid peroxidation; protein carbonyls; Caspase-3 activity; Nrf2 pathway activation.	[87]
	-	In vitro	Primary neurons	Neuroprotective	Suppression/inhibition MPP+ induced: - Apoptosis (↑ Bcl-2/↓ Bax/Caspase-3) - Mitochondrial Dysfunction - Astrocyte Activation (GFAP expression) - Oxidative stress (↓ ROS production) - NF-κB Signaling Pathway.	[88]
Evernic acid	-	In vivo	MPTP-induced mouse model C57BL/6 mice Rotarod	Neuroprotective	Attenuation of motor dysfunction Reduction in dopaminergic neuronal death and astroglial activation.	[88]
	*Evernia prunastri* (L.) Ach.	In vitro	MM98 (malignant mesothelioma cell line) A431 (vulvar carcinoma cell line) HaCaT (human keratinocyte cell line)	Wound healing	No wound closure effects.	[89]
Isolecanoric acid	*Glarea lozoyensis*	In vitro	SH-SY5Y (human dopaminergic neuroblastoma cell line) L-BMAA for amyotrophic lateral sclerosis (ALS) model and rotenone for Parkinson’s disease (PD) model	Neuroprotective	GSK3β and CK1 inhibition. ↓ Oxidative stress, mitochondrial damage, apoptosis, and cell death.	[93]
Lecanoric acid	-	In vitro	α-Glucosidase	Antidiabetic	Active against α-glucosidase (85.9% of inhibition; IC_50_ value of 350 µM)	[98]
	*Umbilicaria ntárctica* Frey and I. M. Lamb.	In vitro	PTP1B enzyme activity and kinetic analysis	Antidiabetic Antiobesity	Moderate inhibition PTP1B activity IC_50_ 31 μM.	[99]
	*Melanelia subaurifera (Nyl.) Melanelia fuliginosa (Fr. Ex Duby) Ess*	In vitro	Gram-positive bacteria: *Bacillus cereus, Bacillus subtilis, Staphylococcus aureus.* Gram-negative bacteria: *Escherichia coli, Proteus mirabilis* Fungi: *Aspergillus flavus, Candida albicans, Mucor. mucedo, Trichoderma viride, Cladosporium cladosporioides, Fusarium oxysporum*	Antimicrobial	Antimicrobial activity against all tested bacteria and fungi with MIC values of 0.5 to 1 mg/mL.	[24]
Lecanoric acid	*Parmelia cetrata Ach.*	In vitro	Gram-negative bacteria: *Aliivibrio fischeri* Nematode *Caenorhabditis elegans*	Antimicrobial Antihelmintic	Antibacterial activity (100% inhibition at 100 µM). Antihelmintic effect (80% mortality at 100 µg/mL).	[4]
	*Melanelia subaurifera (Nyl.) Melanelia fuliginosa* (Fr. ex Duby) Ess	In vitro	DPPH assay	Antioxidant	Slight DPPH scavenging activity (IC_50_ value of 424.5 μg/mL) and reducing power (0.0165 at 125 μg/mL).	[24]
	*Parmotrema grayanum* (Hue) Hale.	In vitro	Superoxide radical (SOR) Nitric oxide radical DPPH assay	Antioxidant	Good antioxidant activity: SOR assay (IC_50_ value = 91.5 µmol), DPPH (IC_50_ value = 34 µmol), NOR assay (IC_50_ value = 53.5 µmol).	[94]
	*Parmotrema stuppeum* (Nyl.) Hale	In vitro	Beta-carotene-linoleate model system	Antioxidant	Thirty-six percent of antioxidant activity at 500 µg/ml.	[48]
	*Hypocenomyce scalaris* (Ach. ex. Lilj	In vitro	Colorectal cancer cells (HCT116 and DLD-1) Human keratinocytes HaCaT cell line	Cytotoxic	Moderate cytotoxic effects against colon HCT116 cells. ↓ Slight Axin2 expression in HCT116 cells.	[95]
	*Parmotrema tinctorum* (Despr. ex Nyl.) Hale.	In vitro	Hep-2 (human larynx carcinoma cells) MCF7 (human breast carcinoma cells) 786-0 (human kidney carcinoma cells) B16-F10 (murine melanoma cells)	Cytotoxic	Slight activity against all tested cancer cell lines (IC_50_ values > 50 µg/mL).	[97]
	*Melanelia subaurifera* (Nyl.) *Melanelia fuliginosa* (Fr. ex Duby) Ess	In vitro	Hela (human epithelial carcinoma cells) A549 (human lung carcinoma cells) LS174 (human colon carcinoma cells)	Cytotoxic	Weak cytotoxic activity against Hela cells (IC_50_ value of 124 μg/mL) and against A549 and LS174 cells (IC_50_ value of 200 μg/mL).	[24]
Lecanoric acid	*-*	In vitro	HCT-116 (human colon cancer cell line)	Cytotoxic	Inhibition cell colony formation already at 0.03 μg/mL. Induction of a G2 cell cycle block. Arrest of cells in the M phase. Upregulated expression of cyclin B1 and pH3. Inactive CDK1. More cell death in cancer cells than in primary human immune and endothelial cells.	[96]
	*-*	In vitro	Mitochondrial TrxR from rat lung	Cytotoxic	High inhibitory effect on Thioredoxin reductase (TrxR).	[79]
Methyl evernate	*Ramalina fastigiata* (Pers.) Ach.	In vitro	Gram-positive bacteria: *Bacillus cereus, Staphylococcus aureus.* Gram-negative bacteria: *Escherichia coli*, *Proteus mirabilis* Fungi: *Aspergillus flavus, Candida albicans, Mucor mucedo, Trichoderma viride, Cladosporium cladosporioides, Fusarium oxysporum, Alternaria alternata, Penicillium expansum*	Antimicrobial	Inhibition against all tested microorganisms. MIC values (from 0.125 to 1 mg/mL).	[24]
	*Ramalina fastigiata* (Pers.) Ach.	In vitro	DPPH assay Reducing power assay	Antioxidant	Low DPPH radical scavenging activity (IC_50_ value of 391.57 μg/mL). Isolated components showed higher reducing power than lichen extracts.	[24]
	*Ramalina fastigiata* (Pers.) Ach.	In vitro	Hela (human epithelial carcinoma cells) A549 (human lung carcinoma cell line) LS174 (human colon carcinoma cells)	Cytotoxic	IC_50_ values of 46.45 μg/mL (Hela cell line), 76.84 μg/mL (A549 cell line), and 161.37 μg/mL (LS174 cell line).	[24]
Olivetoric acid	*Pseudevernia furfuracea var. ceratea* (Ach.) D. Hawksw.	In vitro	RATECs (rat adipose tissue endothelial cells)	Anti-angiogenic	↓ Proliferation. Disruption of endothelial tube formation. Depolymerization effects on F-actin stress fibers.	[104]
	*Pseudevernia furfuracea var. ceratea* (Ach.) D. Hawksw.	In vitro	Gram-positive bacteria: *Staphylococcus aureus, Bacillus cereus, Bacillus subtilis, Streptococcus faecalis, Listeria monocytogenes.* Gram-negative bacteria: *Escherichia coli, Pseudomonas aeruginosa, Pseudomonas vulgaris, Yersinia enterocolitica, Aeromonas hydrophila, Pseudomonas syringae, Klebsiella pneumoniae, Salmonella typhimurium.* Fungi: *Aspergillus niger, Penicillium notatum, Fusarium solani, Fusarium moniliforme, Fusarium oxysporum, Fusarium. culmorum, Candida albicans, C.glabrata, Alternaria. tenuissima, A. citri, A. alternata, Gaeumannomyces graminis.*	Antimicrobial	Active against all bacteria and yeast except *K. pneumoniae*, *P. aeruginosa*, and *P. syringae*. Active against all tested fungi except *A. citri*, A. *tenuissima, A.niger*, and *G. graminis*.	[100]
	*Pseudevernia furfuracea* (L.) Zopf	In vitro	Cultured human amnion fibroblasts	Antioxidant	↓ Cell viability (IC_50_ values of 571.27 mg/mL) <50 mg/L no oxidative stress and genotoxicity.	[101]
	*Pseudevernia furfuracea* (L.) Zopf	In vitro	HLs (cultured human lymphocytes)	Antioxidant	↑ Total antioxidant capacity.	[16]
Olivetoric acid	*Pseudevernia furfuracea* (L.) Zopf	In vitro	U87MG (glioblastoma multiforme cell line) PRCC cells (neurons from Sprague Dawley^®^ rats)	Cytotoxic	↓ Cell viability (IC_50_ values of 125.71 mg/mL, for PRCC cells and 17.55 mg/L for U87MG cells). ↑ 8-OH-dG levels. LDH activity and oxidative DNA damage.	[102]
	*Pseudevernia furfuracea* (L.) Zopf	In vitro	HepG2 (human hepatocellular carcinoma cells)	Cytotoxic	Cytotoxicity with 100–400 mg/L. Upregulation of pro-apoptotic genes (BAK, CASP6, CASP7, CASP8, FADD, FAS, FASLG).	[103]
Perlatolic acid	*-*	In silico	Microsomal prostaglandin E2 synthase 1	Anti-inflammatory	Potent inhibitor of microsomal prostaglandin E2 synthase-1 (IC_50_ = 0.43 µM).	[106]
	*Cetrelia monachorum* (Zahlbr.) W.L. Culb. and C.F. Culb.	In vitro In vivo	Stimulated A549 lung epithelial adenocarcinoma cells Stimulated HEK-293 cells Thioglycollate-induced C57BL/6J male murine peritonitis model	Anti-inflammatory	Microsomal prostaglandin E2 synthase-1 inhibition (IC_50_ = 0.4 µM), 5-Lipoxygenase inhibition (IC_50_ = 1.8 µM for cell-based assay and IC_50_ = 0.4 µM for purified enzyme). Tumor necrosis factor alpha-induced NF-kB (IC_50_ = 7 µM). Inhibition of leukocyte recruitment.	[107]
	*Stereocaulon* sp.	In vitro	Methicillin-resistant *Staphylococcus aureus* strains	Antimicrobial	MIC_90_ value of 32 µg/mL. Synergic action with gentamicin and antagonism action with levofloxacin.	[105]
	*Cladina confusa* (Sant.). Folmm and Ahti	In vitro	Cultures of peritoneal macrophage cells from mice	Immune modulating	↑ Hydrogen peroxide release (10.48 nmol). Slight NO release activity.	[108]
	*Cladonia portentosa* (Dufour) Coem.	In vitro Ex vivo	Neuro2A (mouse neuroblastoma) cell line Primary neural stem or progenitor cells	Neuroprotective	Neurotrophic activity (125.34 μm at 0.5 μM). AChE inhibition activity (IC_50_ = 6.8 μM). Potent proneurogenic activity. Gene expression of BDNF and NGF modulation. ↑ Acetyl H3 and H4 protein levels.	[63]
Ramalic acid/Obtusatic acid	*Ramalina fraxinea* (L.) Ach. *Ramalina fastigiata* (Pers.) Ach.	In vitro	Gram-positive bacteria: *Bacillus cereus, Bacillus subtilis, Staphylococcus aureus.* Gram-negative bacteria: *Escherichia coli*, *Proteus mirabilis* Fungi: *Aspergillus flavus, Aspergillus niger, Candida albicans, Mucor mucedo, Trichoderma viride, Cladosporium cladosporioides.*	Antimicrobial	Inhibition against all tested microorganisms. MIC values (from 0.125 to 1 mg/mL).	[24]
Ramalic acid/Obtusatic acid	*Ramalina fraxinea* (L.) Ach. *Ramalina fastigiata* (Pers.) Ach.	In vitro	DPPH assay Reducing power assay	Antioxidant	Slight to moderate antioxidant activity (DPPH radical scavenging activity with IC_50_ value of 324.61 μg/mL and reducing power of 0.0142 at 125 µg/mL). Isolated components showed higher reducing power than lichen extracts.	[24]
	*-*	In vitro	HaCaT (human keratinocyte cell line)	Cytotoxic	No significant inhibitory activity against LTB (4) production via non-mediation by redox reactions. No cytotoxic activity.	[76]
	*Ramalina fraxinea* (L.) Ach. *Ramalina fastigiata* (Pers.) Ach.	In vitro	Hela (human epithelial carcinoma cell line) A549 (human lung carcinoma cell line) LS174 (human colon carcinoma cell line)	Cytotoxic	IC_50_ value (Hela) 43.24 μg/mL; IC_50_ value (A549) 93.98 μg/mL; IC_50_ value (LS174) 74.28 μg/mL.	[24]
Sekikaic acid	*Dirinaria consimilis* (Stirt.) D. D. Awasthi	In vivo	STZ-induced type 2 diabetic albino rat model	Antidiabetic	↑ α-glucosidase and α-amylase inhibition. ↓ Plasma glucose levels (44.17%), low-density. lipoprotein, total cholesterol, and total glycerides.	[111]
	*Ramalina roesleri* Nyl	In vitro	Gram-positive bacteria: *Bacillus subtilis*, *Staphylococcus aureus*, *Streptomyces viridochromogenes*, *Streptococcus mutans.* Gram-negative bacteria: *Escherichia coli*.	Antimicrobial	Maximum antimicrobial activity against *E. coli* (78% inhibition), moderate against *S. mutans*, *S. aureus*, and *S. viridochromogenes* (60%, 50% and 55% inhibition, respectively), and low against *B. subtilis* (15% inhibition).	[109]
Sekikaic acid	*Ramalina farinacea* (L.) Ach	In vitro	Respiratory syncytial virus	Antimicrobial	Potent antiviral action against a recombinant strain rg respiratory syncytial virus (IC_50_ 5.69 µg/mL) and respiratory syncytial virus A2 strain (IC_50_ 7.73 µg/mL).	[110]
	*Ramalina roesleri* Nyl	In vitro	DPPH assay	Antioxidant	Good antioxidant activity: DPPH radical assay (IC_50_ value = 11.24 µg/mL).	[109]
	*Heterodermia obscurata* (Nyl.) Trevisan	In vitro	Superoxide radical (SOR) Nitric oxide radical DPPH assay	Antioxidant	Good antioxidant activity: SOR assay (IC_50_ value = 82.0 µmol), DPPH (IC_50_ value = 32.6 µmol). No nitric oxide radical activity.	[94]
	*Dirinaria consimilis* (Stirt.) D. D. Awasthi	In vitro	Ferric ion reducing power and hydroxyl radical assay.	Antioxidant	Good antioxidant activity: hydroxyl radical assay (IC_50_ value = 41.5 µg/mL) and ferric ion assay (IC_50_ value = 42.0 µg/mL).	[33]
	*Niebla homalea* (Ach.) Rundel and Bowler	In vitro	MCF-7 (human hormone-dependent breast) A2780 (ovarian cancer cell)	Cytotoxic	No antiproliferative activity.	[112]
Squamatic acid	*Cladonia uncialis* (L.) F. H. Wigg.	In vitro	Gram-positive bacteria: *Staphylococcus aureus* Gram negative bacteria: *Escherichia coli*, Fungi: *Candida albicans*	Antimicrobial	Weak antibacterial activity (MIC = 1250.0 mg/mL against *S. aureus*).	[113]
	*Thamnolia vermicularis* (Sw.) Schaer	In vitro	PC-3 (prostate cancer cells)	Cytotoxic	Weak antiproliferative effect.	[114]
Thamnolic acid	*Usnea florida* (L.) F.H. Wigg	In vitro	Gram-positive bacteria: *Bacillus cereus, Bacillus subtilis, Listeria monocytogenes, Staphylococcus aureus, Enterococcus faecalis, Enterobacter aerogenes, Micrococcus luteus.* Gram-negative bacteria: *Escherichia coli, Klebsiella pneumoniae, Pseudomonas aeruginosa, Proteus vulgaris, Salmonella typhimurium, Yersinia enterocolitica.* Mycobacteria: *Mycobacterium tuberculosis.* Fungi: *Candida parapsilosis, Candida albicans, Candida globrata, Aspergillus niger, Aspergillus flavus, Fusarium moniliforme, Rhizopus* sp., *Alternaria brassicola*, *Sclerotium rolfsii*, *Fusarium solani*	Antimicrobial	Antifungal: *Alternaria alternate*, *Aspergillus fumigatus* and *Sclerotium rolfsii* with MIC values of 400, 400, and 200 µg/mL, respectively. Anti-yeast: *Candida krusei* with MIC value of 400 µg/mL. Antibacterial: *Bacillus cereus*, *Bacillus subtilis*, and *Proteus vulgaris* with MIC value of 400 µg/mL and *Listeria monocytogenes* and *Micrococcus luteus* with MIC value of 200 µg/mL.	[115]
	*Thamnolia vermicularis* (Sw.) Schaer.	In vitro	PC-3 (prostate cancer cells)	Cytotoxic	Weak antiproliferative effect.	[114]

### 2.2. Tridepsides

**Figure 2:**
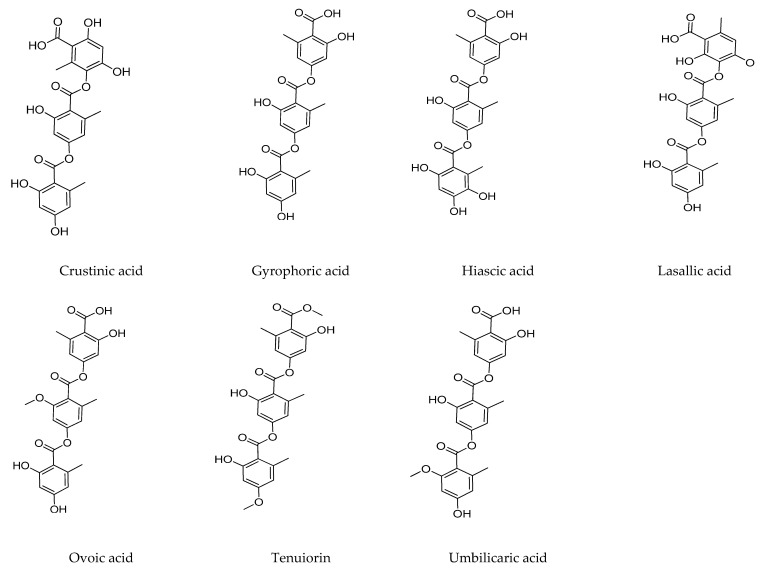
Chemical structure of tridepsides from lichens.

The biological activities and tridepside chemical structures have been gathered in Table 2 and Figure 2.

#### 2.2.1. Gyrophoric Acid

Gyrophoric acid is a potent antimicrobial agent against a wide range of bacteria and fungi, with MIC values from 0.019 mg/mL for *B. subtilis* [117]. Moreover, the antimicrobial activity for this tridepside was also demonstrated by Candan et al., highlighting its effect against the bacteria *Bacillus cereus* and *Bacillus subtilis* and the fungi *Candida albicans* and *Candida glabrata* [118]. Furthermore, gyrophoric acid showed larvicidal activity against the second and third instar larvae of the mosquito *Culiseta longiareolata*, with LC (50) and LC (90) values of 0.41 ppm and 1.93 ppm, respectively [39].

Gyrophoric acid has also demonstrated potent antioxidant properties. Hence, the IC_50_ values for DPPH and superoxide anion scavenging were 105.7 µg/mL and 196.6 µg/mL, respectively, and its reducing power value was 1.32 at 1000 µg/mL [117].

Gyrophoric acid has been investigated for its role as a cytotoxic agent against different cancer cells. This compound reduced the cell viability of human ovarian carcinoma (A2780 cells), human promyelocytic leukemia (HL-60 cells), human T cell lymphocyte leukemia (Jurkat cells), malignant melanoma (Fem-x cells), and chronic myelogenous leukemia (K562 cells) [55,117]. In particular, the studies on A2780 cancer cells revealed that gyrophoric acid caused the accumulation of these cells in the G2/M phase at the expense of the G0/G1 phase. Moreover, this compound reduced the percentage of Fem-x cells and K562 cells in the G0/G1 and S-G2/M phases of the cell cycle [117]. Furthermore, this secondary metabolite inhibited the clonogenic ability of breast SK-BR-3 cancer cells [55]. Additionally, gyrophoric acid caused the cell death of human cervix carcinoma (HeLa) by oxidative stress and apoptosis pathways, as evidenced in the ROS overproduction, DNA oxidative damage, and caspase-3 activation [17]. A study on lichen compounds that interact with DNA revealed that gyrophoric acid was able to inhibit topoisomerase I activity at a concentration of 25 µM [61]. However, this tridepside has resulted in being inactive as an apoptotic agent, as revealed by its ineffectiveness as a caspase-3 activator on hepatocytes [119], and it has low activity against A375 melanoma cancer cell line even at the highest concentrations tested [58].

Other studies, based on the antiproliferative capacity of gyrophoric acid, have investigated its effect on skin cells for therapeutic purposes for psoriasis. This tridepside significantly inhibited the growth of the human keratinocyte HaCaT cell line, with the IC_50_ value of 1.7 µM by a cytostatic mechanism [76]. Moreover, gyrophoric acid exerted a photoprotective effect on HaCaT cells with a sun protection factor (SPF) of (SPF > 5) [120,121]. Anti-aging effects were also investigated on ultraviolet A (UVA)-treated dermal fibroblasts, showing upregulated mRNA levels of COL1A1/COL3A1/SOD2 genes and type I collagen protein levels [122].

Gyrophoric acid has been shown to have antihypertensive properties by acting as an angiotensin II type-1 receptor (AT1) antagonist by interacting with residues ARG167, TRP84, and VAL108 [123]. Moreover, gyrophoric acid has been identified as a non-competitive PTP1B inhibitor, with an IC_50_ value of 3.6 μM, making it a drug candidate for type 2 diabetes and obesity [99]. Finally, gyrophoric acid is of interest for its properties as a healing agent, especially when combined with usnic acid, that promotes tissue regeneration [89].

#### 2.2.2. Tenuiorin Acid

Tenuiorin acid showed weak to moderate antiproliferative action against the human cancer breast T-47D cell line (ED_50_ 152.6 μM), the human cancer colon WIDR cell line (ED_50_ 95.9 μM), and the human cancer pancreas PANC-1 cell line (ED_50_ 87.9 μM), which seems to be related to its ability to inhibit 5-lipoxygenase activity [22]. Using a Thioflavin T (ThT) fluorescence assay, tenuiorin acid was a potent neuroprotective agent which acted as a tau inhibitor (IC_50_ 100 µM) [124].

#### 2.2.3. Trivaric Acid

The tridepside trivaric acid has resulted in being a promising antidiabetic agent. In silico and in vitro studies revealed that this compound inhibited protein tyrosine phosphatase 1B (PTP1B) by blocking its active site with an IC_50_ value of 173 nM. Moreover, this tridepside improved the insulin-stimulated glucose uptake through the insulin receptor (IR)/IRS/Akt/GLUT2 pathway in the human liver HepG2 cancer cell line. Furthermore, in vivo studies demonstrated the beneficial effects of trivaric acid as an antidiabetic agent at doses of 5 mg/kg and 50 mg/kg through significantly improving lipid and glycemic profiles [125,126].

In another study, trivaric acid exerted a potent anti-inflammatory action by significantly inhibiting human leukocyte elastase (IC_50_ value of 1.8 µM) [127].

**Table 2:** Pharmacological activity of lichen tridepsides.

Tridepside	Botanical Origin	Type of Study	Experimental Model	Activities	Results	References
Gyrophoric acid	*Umbilicaria antarctica* Frey and I. M. Lamb.	In vitro	PTP1B enzyme activity and kinetic analysis	Antidiabetic Antiobesity	Inhibition PTP1B activity IC_50_: 3.6 μM in a non-competitive manner.	[99]
	*Parmelia saxatilis* (L.) Ach.	In vitro In silico	Angiotensin II type-1 receptor (AT1) interaction	Antihypertensive	AT1 antagonist. Calcium influx assay (IC_50_ 29.76 μM).	[123]
	*Acarospora fuscata* (Nyl.) Th.Fr.	In vitro	Gram-positive bacteria: *Bacillus mycoides*, *Bacillus subtilis Staphylococcus aureus*. Gram-negative bacteria: *Escherichia coli*, *Klebsiella pneumoniae*. Fungi: *Aspergillus flavus*, *Aspergillus fumigatus*, *Candida albicans*, *Penicillium purpurescens* and *Penicillium verrucosum.*	Antimicrobial	Minimum inhibitory concentration values ranging from 0.019 to 1.25 mg/mL.	[117]
	*Xanthoparmelia pokornyi* (Körb.) O.Blanco, A.Crespo, Elix, D.Hawksw. and Lumbsch	In vitro	Gram-positive bacteria: *Bacillus cereus*, *Bacillus subtilis, Listeria monocytogenes*, *Staphylococcus aureus*. *Streptococcus faecalis*. Gram-negative bacteria: *Aeromonas hydrophila*, *Proteus vulgaris*, *Yersinia enterocolitica*. Fungi: *Candida albicans* and *Candida glabrata*.	Antimicrobial	Active against some bacteria and fungi (*A. hydrophila*, *B. cereus*, *B. subtilis, L. monocytogenes*, *P. vulgaris*, *S. aureus*, *S. faecalis*, *Y. enterocolitica*, *C. albicans* and *C. glabrata*)	[118]
Gyrophoric acid	*Acarospora fuscata* (Nyl.) Th.Fr.	In vitro	DPPH Superoxide anion radical-scavenging reducing power	Antioxidant	DPPH (IC_50_ 105.75 µg/mL). Superoxide anion radical (IC_50_ 196.62 µg/mL). Reducing power (1.32 at 1000 µg/mL, 1.12 at 500 µg/mL, 0.71 at 250 µg/mL, 0.39 at 125 µg/mL, and 0.20 at 62.5 µg/mL).	[117]
	*Parmelia nepalensis* Tayl. *Parmelia* *tinctorum* Nyl.	In vitro	HaCaT (human keratinocyte cell line)	Antiproliferative	Antiproliferative activity (IC_50_ value of 1.7 µM). Cytostatic mechanism.	[76]
	*Umbilicaria hirsuta* (Sw. ex Westr.) Hoffm	In vitro	A2780 (human ovarian carcinoma) HCT-116 p53+/+ and HCT-116 p53−/− (human colon carcinoma) HeLa (human cervix adenocarcinoma) SK-BR-3 (human breast adenocarcinoma) HL-60 (human promyelocytic leukemia) HT-29 (human colon adenocarcinoma) Jurkat (Human T cells lymphocyte leukemia) MCF-7 (Human breast adenocarcinoma)	Cytotoxic	Effective against A2780, HL-60, and Jurkat cells. Clonogenic ability inhibition of SK-BR-3 cells. A2780 cells accumulation in S-phase at expense of G1/G0-phase.	[55]
Gyrophoric acid	*Umbilicaria hirsuta* (Sw. ex Westr.) Hoffm	In vitro	HeLa (human cervix carcinoma)	Cytotoxic	Oxidative stress pathway: ↑ ROS level, DNA oxidation and activity changes of stress/survival proteins as p38MAPK, Erk1/2 and Akt. Apoptosis pathway: ↑ caspase-3 activation, PARP cleavage, PS externalization, and cell cycle changes.	[17]
	*Acarospora fuscata* (Nyl.) Th.Fr.	In vitro	A549 (human lung carcinoma cell line), Fem-x (malignant melanoma cell line), K562 (chronic myelogeneous leukemia cell line) LS174 (human colon carcinoma cell line)	Cytotoxic	Weak activity against A549 and LS174 (IC_50_ 151.51 and 151.65 µg/mL). Moderate cytotoxic effect against Fem-x and K562 cells (IC_50_ 64.01 and 78.45 µg/mL). Apoptosis of sub-G1 phase in malignant cells. Reduction percentage of cells in G0/G1 and S-G2/Mphases of the cell cycle.	[117]
	*Ochrolechia deceptionis* Hue.	In vitro	A375 (melanoma cancer cell line)	Cytotoxic	Low activity.	[58]
	*-*	In vitro	Primary cultures of rat hepatocytes	Cytotoxic	Inactive.	[119]
	*Umbilicaria hirsuta* (Sw. ex Westr.) Hoffm	In vitro	Calf thymus DNA	DNA-interacting agents	Topoisomerase I inhibition (25 μM).	[61]
	*-*	In vitro	Second and third instar larvae of the mosquito *Culiseta longiareolata*	Larvicidal activity	LC (50) values: 0.41 ppm, LC (90) values: 1.93 ppm.	[39]
	*Xanthoparmelia pokornyi* (Körb.) O.Blanco, A.Crespo, Elix, D.Hawksw. and Lumbsch	In vitro	HaCaT (human keratinocyte cell line)	Photoprotective	Prevention of cytotoxic, apoptotic, and cytoskeleton alterative effects of 2.5 J/cm^2^ UVB.	[121]
Gyrophoric acid	*Lasallia pustulata* (L.) Méra	In vitro	DPPH assay NBT assay Human keratinocytes HaCaT cell line	Photoprotective	DPPH (IC_50_ 25 µg/mL) Good PF-UVA candidate (SPF > 5).	[120]
	*-*	In vitro	(UVA)-treated dermal fibroblasts	Photoprotective	Anti-aging effects. Upregulated mRNA levels of COL1A1/COL3A1/SOD2 genes and type I collagen protein levels. ↓MMP1 mRNA and protein expression levels.	[122]
	*Lasallia pustulata* (L.) Méra	In vitro	MM98 (Malignant mesothelioma cell line) A431 (vulvar carcinoma cell line) HaCaT (human keratinocyte cell line)	Wound healing	Strong wound closure effects. Better results combined with (+)-usnic acid.	[89]
Tenuiorin acid	*Peltigera leucophlebia* (Nyl.) Gyeln.	In vitro	Calcium-stimulated porcine leucocytes. T-47D (human cancer breast cell line) WIDR (human cancer colon cell line) PANC-1 (human cancer pancreas cell line)	Antiproliferative	Moderate 5-lipoxygenase inhibition (IC_50_ values of 41.6 μM). Moderate/weak antiproliferative effects on PANC-1 and WIDR cells (ED_50_ 87.9 and 95.9 μM, respectively) and weak activity against T-47D cells (ED_50_ 152.6 μM).	[22]
	*Umbilicaria antarctica* Frey and I. M. Lamb.	In vitro In silico	ThT fluorescence assay Docking studies	Neuroprotective	Tau inhibitor (IC_50_ 100 µM).	[124]
Trivaric acid	*-*	In silico	Docking and ITC studies	Antidiabetic	PTP1b inhibition by blocking its active site.	[126]
	*-*	In vitro	PTP1b inhibition assay Human liver HepG2 cancer cell line	Antidiabetic	↑ PTP1B inhibitory activity. IR/IRS/Akt/GLUT2 pathway stimulation. ↑ Glucose consumption	[125]
Trivaric acid	*-*	In vivo	Diabetic mice model	Antidiabetic	↓ insulin resistance ↓ leptin resistance. Improve lipid profile and weight control.	[125]
	*-*	In vitro	Human leukocyte elastase assay	Anti-inflammatory	Potent human leukocyte elastase inhibitory activity (IC_50_ of 1.8 µM).	[127]

## 3. Conclusions and Prospects

Most of the works on the pharmacological activity of depsides and tridepsides have been published in the last 10 years, which shows the growing therapeutic interest in the secondary metabolites of lichens. Most of these works are in vitro studies, with the occasional in silico and in vivo studies.

Lichens have been investigated for their ability to inhibit bacterial growth. The most common Gram-positive bacteria genera studied on depsides and tridepsides are *Bacillus* and *Staphylococcus*, followed by *Mycobacterium, Streptococcus*, and *Enterococcus*. Among the Gram-negative bacteria, the genera *Escherichia* and *Proteus* were the most investigated. In general terms, depsides and tridepsides showed weak to moderate antimicrobial activity, being more potent against Gram-positive bacteria. In addition, the antifungal activity of several of these compounds (e.g., atranorin, divaricatic acid, gyrophoric acid, lecanoric acid, and methyl evernate) has mainly been studied against *Candida* spp. Moreover, some depsides displayed good antiparasitic activity against *Plasmodium falciparum* and *Schistosoma mansoni*.

Moreover, antioxidant activity has also been widely investigated using different in vitro techniques, such as DPPH assay, hydroxyl radical scavenging activity, superoxide radical scavenging activity, and reducing power assay. Depsides and tridepsides have a phenolic structure that provides antioxidant properties. The compounds with the greatest capacity to scavenge free radicals are sekikaic acid and atranorin.

The cytotoxic activity of depsides and tridepsides has been extensively investigated in in vitro studies. The mechanisms involved in the cytotoxicity of these compounds imply oxidative stress induction (ROS overproduction), apoptosis induction (caspase-3 activation, Bcl2/Bax signaling pathway), cell-cycle arrest, and 5-lipoxygenase antagonist therapy.

Despite the interesting activities that have been compiled in this review, the information seems to stop after the in vitro assays with diverse cell lines. Only a few studies have continued with the in vivo model. The great variety of activities indicate low specificity, which must be deepened with regard to structure–activity studies and toxicological studies. Preclinical and clinical studies should focus on identifying the molecular targets for the action and the nontoxic doses in humans. Furthermore, there are also several compounds for which there is no study of pharmacological activity, such as hiascic acid, lassalic acid, ovoic acid, crustinic acid, and hypothamnolic acid, all being potential metabolites to be investigated. On the other hand, new technology advances will allow the improvement of growing yields and compound extraction, solving the current problem that limits the study with these interesting compounds

## Data Availability

Not applicable.
